# Exploring the Effectiveness, Tolerability, and Safety of the Adjunctive Use of Microneedling With Tranexamic Acid in the Treatment of Melasma

**DOI:** 10.1111/jocd.70763

**Published:** 2026-02-22

**Authors:** Sharon Dhaliwal, Navneet Dhanoa, Zaina Rashid

**Affiliations:** ^1^ Midwestern University Arizona College of Osteopathic Medicine Glendale Arizona USA; ^2^ La Peau Dermatology Mesa Arizona USA

**Keywords:** adjunctive therapy, hyperpigmentation, melasma, microneedling, tranexamic acid

## Abstract

**Background:**

Melasma is a chronic pigment disorder associated with significant psychosocial burden. The gold standard triple combination cream is effective but limited by relapse and adverse effects. Tranexamic acid (TXA) and microneedling have independently shown promise in the treatment of melasma, and their combined use may provide enhanced efficacy, durability, and tolerability.

**Aims:**

This review aims to synthesize and compare clinical evidence assessing the effectiveness, safety, patient satisfaction, and durability of microneedling‐based TXA for the treatment of melasma.

**Methods:**

A PubMed search was performed for relevant studies published in the last 10 years. Eligible articles included clinical trials, observational studies, and case reports. A scoping literature review was then conducted to synthesize relevant findings.

**Results:**

Most identified studies demonstrated improvement in melasma scores when TXA was delivered through microneedling. Outcomes are comparable to other TXA delivery methods and adjunctive therapies, with microneedling providing potential advantages in satisfaction, tolerability, and durability. These findings support its consideration for patients unable to tolerate systemic TXA or those seeking minimally invasive alternatives to laser‐based treatments.

**Conclusion:**

This review compares microneedling‐based TXA delivery with other treatment approaches, highlighting patient‐centered advantages in tolerability and durability that may inform individualized treatment selection. Future studies should address limitations in protocol heterogeneity, reliance on semi‐objective outcome measures, and limited long‐term follow‐up. Particular focus is needed on evaluating patients with skin of color, who may be more susceptible to post‐inflammatory hyperpigmentation.

## 1 Introduction

Melasma is a skin disorder that manifests with patches of brownish‐gray hyperpigmentation, commonly on sun‐exposed areas of the face. It classically occurs in women of reproductive age, especially those with Fitzpatrick skin types III–V [1]. Despite its widespread prevalence and psychological burden, its pathogenesis and optimal treatment remain incompletely understood [2, 3, 4]. Proposed contributing factors include reproductive hormones, pregnancy, sun exposure, genetics, inflammation, topical corticosteroids, and photosensitive medications [1]. Rather than a disorder solely of melanocyte dysfunction, melasma appears to involve complex vascular, inflammatory, photosensitive, and endocrine alterations. The current gold‐standard therapy is a triple combination cream containing 4% hydroquinone, 0.01% fluocinolone acetonide, and 0.05% tretinoin, derived from Kligman's original formula [5]. However, its adverse effects, including skin atrophy, ochronosis, telangiectasia, pruritus, erythema, and post‐inflammatory hyperpigmentation, along with limited durability of response, have driven the pursuit of safer and more effective alternatives [5, 6, 7].

Tranexamic acid (TXA), an antifibrinolytic agent that inhibits plasmin, has emerged as a promising therapeutic option. Although its precise mechanism in melasma is not fully understood, TXA may reduce melanocyte overstimulation by inhibiting tyrosinase activity and melanocyte‐keratinocyte interactions, as well as lessen neovascularization and dermal inflammation by reducing basic fibroblast growth factor and mast cell activity [5, 8]. Oral TXA can cause nausea, abdominal pain, diarrhea, and oligomenorrhea [5, 9]. With a more favorable cutaneous side effect profile and multiple delivery modalities (oral, topical, intradermal microinjection, and mesoneedling), TXA has become a versatile treatment option for melasma [5, 9].

Microneedling has also become widely used in melasma management. It creates microchannels in the skin with fine needles and may help regulate increased melanocyte stimulation and vascular abnormalities [10, 11]. Its primary mechanism is microtrauma induced activation of wound healing pathways, resulting in increased collagen and elastin production and epidermal thickening [10, 11, 12]. Microneedling also facilitates enhanced delivery of topical agents, known as mesoneedling [10]. Side effects are generally mild and transient, including erythema, burning, and pruritus [10, 11]. Current literature supports microneedling as an effective adjuvant therapy in melasma when combined with depigmentation agents such as vitamin C, TXA, and hydroquinone [10].

Several clinical studies have examined the use of TXA delivered via microneedling for the treatment of melasma; however, these studies are heterogeneous in design, delivery method, comparator therapy, and outcome measures. To date, no review has synthesized the results of these studies with a focus on treatment effectiveness, safety, tolerability, and durability of outcomes. This scoping review aims to consolidate and summarize the available clinical evidence to support informed and individualized treatment decision making in melasma management.

## 2 Methods

The scoping review was performed using the PubMed search engine and the following Boolean algorithm: melasma AND (microneedling OR “micro needling” OR mesoneedling OR “percutaneous collagen induction” OR dermaroller OR dermapen). The articles were obtained in PubMed using the following filters: in the last 10 years, Case Reports, Clinical Study, Clinical Trial, Clinical Trial Phase I, Clinical Trial Phase II, Clinical Trial Phase III, Clinical Trial Phase IV, Clinical Trial Protocol, Controlled Clinical Trial, Observational Study, Randomized Controlled Trial. Articles were excluded from the scoping review on the basis of the criteria: irrelevant to the objective of review, unavailable in English text, review articles, animal only studies, publication greater than 10 years ago, and inability to retrieve a free, online full text article.

## 3 Results

### 3.1 Literature Search

**FIGURE 1:**
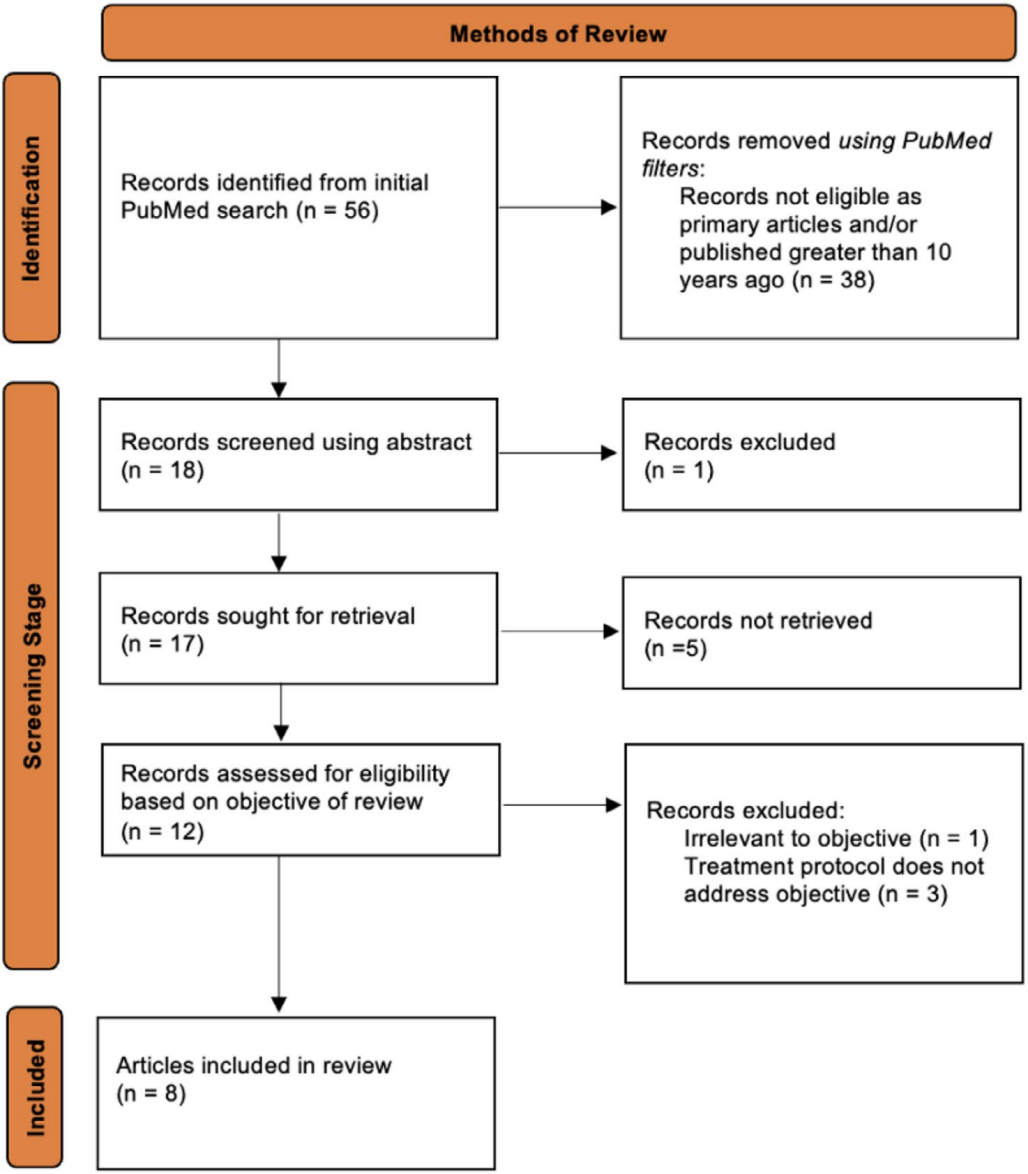
PRISMA flow diagram depicting screening process used in scoping review.

Fifty‐six articles were initially identified using the PubMed search engine. Through a systematic screening process, eight articles were ultimately selected for review (Figure 1). These studies evaluated the adjunctive use of microneedling with TXA in the treatment of facial melasma (Table 1).

**TABLE 1:** Description of the characteristics, purpose, methods, and key findings of the eight articles included in the scoping review.

Authors	Article title	Article type	Purpose	Methods	Key findings
Raza et al.	Split‐Face Comparative Analysis Of Micro‐Needling With Tranexamic Acid Vs. Vitamin C Serum In Melasma	Controlled clinical trial	Evaluation of differences in efficacy of topical tranexamic acid compared to vitamin c serum via microneedling in treating facial melasma	30 patients (19 females, 11 males) with symmetrical facial melasma in Pakistan, aged 20–55—Approx 90% patients were Fitzpatrick skin type IV—Split face design: Each pt. received TXA via microneedling on right hemiface and vitamin C on left hemiface—3 sessions occurring every 2 weeks—Measured outcomes at 2, 4, and 6 weeks time points using physician and patient global assessment scores, mMASI.—Improvement stratification based on global assessments: Poor = 0%–25%, Fair = 25%–50%, Good = 50%–75%, Excellent ≥ 75%	Significantly decreased melasma severity in TXA treated—but not vitamin c treated—side in 27% patients following one session, at 2 weeks follow up (*p* < 0.05) Following 3 treatments (after 6 weeks), mMASI scores improved significantly in both TXA and vitamin c groups (*p* < 0.01). No difference between TXA and vitamin C responses at this time point (*p* > 0.05) Patient and physician global assessments mean scores revealed similar improvements in TXA and Vitamin C treated sides after 1 session: Vitamin C hemiface: 13% excellent response, 43% good, 30% fair TXA hemiface: 16% excellent, 40% good, 26% fair Patients with deep dermal melasma that was widespread had negligible improvement in mMASI scores on either TXA or vitamin c treated sides
Ebrahim et al.	Tranexamic Acid for Melasma Treatment: A Split‐Face Study	Controlled clinical trial	Evaluating the therapeutic response and safety of TXA delivered via intradermal injection versus micro needling in the tx of melasma	56 female patients with symmetrical facial melasma. Fitzpatrik skin types III‐IV. Aged 27–50 years. Conducted in Egypt.—Split‐face study: one side received micro needling with TXA (6 sessions, 2 weeks intervals) and the other a one‐time intradermal TXA injection—Baseline and post treatments photos—Measured efficacy with mMASI score at baseline and after tx—Patient self assessment and satisfaction scores obtained post tx	Both intradermal and micro needling treatments yielded reduced mMASI score from baseline following the treatment protocol (*p* < 0.001) 13.83 ± 7.23 to 3.49 ± 2.91 on intradermal side and 13.83 ± 7.23 to 3.65 ± 2.32 on microneedling side Percent change for intradermal side = 74.8% Percent change for micro needling side = 73.6% No difference between treated sides (*p* > 0.05) Patient reported satisfaction higher with micro needling treatment compared to intradermal injection (*p* < 0.001) Limited adverse effects Minor erythema and edema in both sides (45%) Injection site pain with intradermal tx (43%) Minor irritation with micro needling tx (20%) 16% melasma recurrence in both treatment arms
Poostiyan et al.	Tranexamic Acid Microinjections Versus Tranexamic Acid Mesoneedling in the Treatment of Facial Melasma: A Randomized Assessor‐Blind Split‐Face Controlled Trial	Investigator‐blinded, randomized controlled trial	A comparison of the efficacy and safety of TXA microinjections and TXA mesoneeedling in the treatment of facial melasma	27 female patients in Iran with symmetrical facial melasma. Average age 44.22 ± 8.39 years. Fitzpatrick skin scale II‐V.—Examiner‐blind and split‐face study—One hemiface subjected to 100 mg/mL TXA mesoneedling, other hemiface received TXA microinjections intradermally (identical TXA solution—0.1 mL, injected at 1 cm intervals) 3 total treatments at Weeks 0, 4, and 8. Primary dependent variable: mMASI score improvement 4 weeks following the last treatment compared to baseline Other outcomes: adverse effects and pt. satisfaction by use of visual analog scale (VAS)	Mesoneedling (topical TXA via microneedling) and TXA microinjections yielded similar efficacy Standardized mean difference, SMD, of mMASI scores at baseline: 0.32 [95% CI:‐0.22, 0.85] *p* = 0.248 After final treatment: −0.13 [95% CI: −0.66, 0.40] *p* = 0.633 Change in mMASI scores pre and post treatment were not statistically different among the mesoneedling versus microinjection groups: −0.39 [95% CI: −0.93, 0.15], *p* = 0.157 TXA mesoneedling yielded greater patient satisfaction than microinjection as determined by VAS: SMD = 0.77 [95% CI: 0.21, 1.32] *p* = 0.007 Adverse effects: Mesoneedling with TXA group had higher prevalence of scaling, erythema, and edema (*p* < 0.001) and one case of post‐inflammatory hyperpigmentation
Debasmita et al.	A Prospective Randomized Controlled Trial of Q‐Switched Nd:YAG Laser With Topical 3% Tranexamic Acid (TA) Versus Microneedling With Topical 3% Tranexamic Acid (TA) in Treatment of Melasma	Prospective, randomized controlled clinical trial	Compare the efficacy and side effects of microneedling and QS Nd:YAG laser when combined with daily topical 3% TXA gel	Participants: 60 patients 18–50‐year‐old with varying severity of melasma, 5 sessions, follow up after 2 months. Study duration of 1 year. Group A: Nd:YAG laser (Alma rejuve, 1064 mm, fluence 800 mJ/cm2, 4 Hz freq, 2.5 and 4 mm spot size) sessions monthly with daily 3% TA gel at night Group B: Microneedling (dermaroller 1.5 mm) monthly with daily TA gel at night (provided topical eutectic mixture of local anesthetics (EMLA)) Inclusion Criteria: untreated cases, age 18–50 with melasma of more than 3 months Exclusion criteria: Patients on photosensitizing drugs, hormonal therapy, hx keloid formation, herpes, collagen vascular disease, known allergy to TA, known bleeding diathesis. Dependent variables: mMASI, patient satisfaction scores, and photography at baseline, 3rd session, 5th session, and final follow up	56 patients completed trial (2 patients in each group lost to follow up—reason unknown). Mean decrease in mMASI at follow‐up in Group A 5.12 ± 2.66 to 2.33 ± 1.33, Group B was 4.60 ± 2.38 to 1.88 ± 1.08 (*p* < 0.001). Comparative decrease in mMASI in both groups NOT statistically significant (*p* = 0.14), but precentage decrease was 55% in Group A and 60% in group B. Patient satisfaction scores not significantly different. Side effects of both interventions included erythema, pain, burning sensation, however, side effects of pain and erythema were considerably higher in microneedling group. Study showed equal efficacy of microneeding and QS Nd:YAG laser when combined with topical 3% TA gel in treating melasma without serious side effects. Microneedling entails more downtime
Sharma et al.	Therapeutic Efficacy and Safety of Oral Tranexamic Acid and That of Tranexamic Acid Local Infiltration With Microinjections in Patients With Melasma: A Comparative Study	Randomized clinical trial	Ascertain the comparative efficacy of different routes of administration of TXA	100 patients with melasma (8 men, 92 women) 18–55‐year‐old, Fitzpatrick skin type IV, Indian population Group A: oral TXA 250 mg twice daily Group B: intradermal microinjections of TXA 4 mg/mL every 4 weeks—All patients were evaluated at baseline and every 4 weeks (0, 4, 8, 12, 24) until end of study period for therapeutic outcome. 12 weeks treatment period. Percentage reduction in baseline MASI was assessed at 4 weeks intervals and response was scored (very good: > 75% reduction, good: 50%–75%, moderate: 25%–50%, mild: < 25%)	Study was completed by 39 patients in group A and 41 patients in group B Very good response was seen in 25 and 32 patients in Groups A and B, while good response was seen in 14 and 9 patients, respectively. Both treatment methods were equally effective, with average reduction of MASI at 12 weeks of 77.96 ± 9.39 in Group A and 79.00 ± 9.64 in Group B TXA appears to be an effective and safe treatment for melasma, irrespective of its route of administration 2 patients in group A had relapsed at 24 weeks. No relapses in Group B. Adverse effects: epigastric discomfort, hypomenorrhea, headache, injection site pain = did not warrant discontinuation of treatment. Limitations: small number of patients, no control arm, short duration of treatment and follow up, use of MASI (subjective assessment tool), no objective assessment of overall post‐treatment satisfaction
Arida et al.	Randomized, Double‐Blind, Placebo‐Controlled Split‐Face Trial of the Efficacy of Tranexamic Acid by Drug Delivery Through Microneedling in the Treatment of Melasma	Randomized, double blind, self‐controlled trial	Evaluate efficacy of tranexamic acid while applied by drug delivery through microneedling in the treatment of facial melasma	Split face trial with 3 monthly sessions in 20 female melasma patients older than 18 years (avg age 42) without treatment for at least 6 months. FItzpatrick types II‐V. 16 weeks duration. Protocol: Microneedling (1.5 mm polyethylene roll) was performed in the entire face, then TA solution (1 mL of 50 mg/mL TA) was applied to one hemiface and placebo (0.9% saline) to the other. Effectiveness was measured using Hemi‐MASI, images pixels, and perceptions of experts and patients After first assessment, standardized triple formula of 0.5% retinoic acid, 4% hydroquinone, and 0.01% flucinolone acetonide (hormoskin) for topical use every night for 30 days before first procedure and standardized sunscreen with SPF 50. Instructed to stop using formula 48 h before the procedure. 30 days after first assessment, received 3 procedures with 30‐day interval between sessions. 7.5 g topical anesthetic 4% lidocaine applied to entire face at each session and reapplied 30 min after. After procedure, patients instructed to not wash face for next 6 h, use sunscreen the next day, apply regenerating cream (Cicaplast baume) twice a day for the first 2 days and, after 48 h, return the use of the triple formula. 30‐day after third procedure, last evaluation. Exclusion criteria: pregnant, breastfeeding, severe comorbidities, coagulation disorders, use of anticoagulants, healing problems, presence of dermatitis or current infection on face, disease with possibility of developing Koebner phenomenon, low capacity to understand terms of study	Hemi‐MASI regressed 22% in control and 29% in TA side (*p* = 0.52). Good/better improvement was found in 37.5% of control and 42.5% of TA by experts, and 60% of patients for both sides. Pixels increased by 5 and 7 None of these criteria had a significant difference btween the sides. TXA in drug delivery through microneedling did not bring additional benefits to the treatment of melasma
Cassiano et al.	Efficacy and Safety of Microneedling and Oral Tranexamic Acid in the Treatment of Facial Melasma in Women: An Open, Evaluator‐Blinded, Randomized Clinical Trial	Investigator‐blinded, randomized clinical trial	Efficacy of TXA and microneedling as adjuvants to the standard treatment of melasma	Randomly assigned 64 women (aged > 18) with facial melasma without treatment for 1 month to 4 groups. Conducted in Brazil. M Group: 2 sessions (at inclusion and after 30 days) of microneedling (1.5 mm) under topical anesthesia + took placebo orally 2× daily for 60 days T Group: oral TA 250 mg 2×/day for 60 days MT Group: both oral TA + 2 sessions of microneedling CT Group: no microneedling + took placebo for 60 days All participants required to use broad spectrum sunscreen SPF 50 during day and triple combination cream (Tri‐Luma) at night in combination with the interventions for 60 days, followed by maintenance through Day 120. Assessed via Modified MASI (mMASI) score at 30, 60, and 120 days. Melasma Quality of Life Scale and difference between colorimetric luminosity from perilesional skin to the melasma (DifL) were also evaluated	All groups showed reduction in mMASI at 30 and 60 days. MT and T groups showed early (Day 30) improvement superior to the CT group (*p* < 0.03) At 60 days, M, T, and MT groups performed better than CT group (*p* < 0.05). No superiority in the MT group compared with M and T groups (*p* > 0.1). At maintenance follow‐up (Day 120), T group performed worse than the CT group (*p* = 0.04). At D120, no difference between the M and MT groups (*p* = 0.47) All groups showed improvements in quality of life scores, but M and MT groups presented early (Day 30) results. DifL analysis showed early decrease in MT group but revealed superiority of T, M, and MT groups over the CT group at Day 60. Adverse effects: Oral TA: nausea, abdominal pain, hair loss, blurred vision. One patient had to stop oral TA due to persistance headaches. Microneedling: one episode of herpes simplex occurred in 3 patients, treated with acyclovir. Microneedling and TA both improved performance of triple‐combination cream. Interventions that used microneedling seemed to promote lower relapse. Both TA and microneedling contribute to early clinical response, while microneedling provides sustained remission over the standard therapy
Xu et al.	Efficacy of Functional Microarray of Microneedles Combined With Topical Tranexamic Acid for Melasma: A Randomized, Self‐Controlled, Split‐Face Study	Randomized, controlled clinical trial	Evaluate efficacy of a functional microarray of microneedles plus topical tranexamic acid for melasma in middle aged women in China	30 female subjects with melasma, age 20–50 years, Fitzpatrick skin types III‐IV. Conducted in China. Protocol: one side of face pretreated with functional microarray of MNs, followed by topical 0.5% TA solution 1×/week for 12 weeks. Other side of face (control) treated with sham device plus topical 0.5% TA solution. Dependent variables: Clinical evaluations (photographic) and parameters determined by VISIA were recorded at baseline and Weeks 4, 8, 12 of treatment. At baseline and Week 12, patient satisfaction scores and biophysical parameters were measured by Mexameter	28 women completed the study Brown spots' scores measured by VISIA were significantly lower on the combined therapy side than control at 12 weeks. No significant difference between sides at 4 or 8 weeks. After 12 weeks, melanin index (MI) decreased significantly in both groups, and MI was significantly less on combined side at Week 12. Physicians evaluations of photographs showed better results at Week 12 with combined therapy (> 25% improvement was observed in 25 pts. on combined therapy, and only 10 patients on TA only). Subjective satisfaction scores increased significantly on both sides. Patients were more satisfied with results of combined therapy side than control. Transepidermal water loss, roughness, skin hydration, skin elasticity, and erythema index showed no significant difference between sides at baseline, 2, 8, 12 weeks. No obvious adverse events

### 3.2 Study Objectives and Design

Eight prospective, controlled clinical trials evaluating the safety and efficacy of microneedling‐based TXA delivery for melasma were included in this scoping review. The most common study duration was 12 weeks, with follow‐up ranging from 6 to 24 weeks. All but three studies used randomized or nonrandomized split‐face designs, allowing each participant to serve as their own control [13, 14, 15, 16, 17, 18, 19, 20]. Poostiyan et al. employed an investigator blind design and Arida et al. conducted a double‐blind trial [13, 14]. Routes of TXA administration included microneedling/mesoneedling, intradermal microinjections, and microarray microneedling. Raza et al. uniquely compared microneedling with TXA versus vitamin C serum [15], while Debasmita et al. examined TXA combined with microneedling versus Q‐switched Nd:YAG laser [16].

### 3.3 Demographics

Sample sizes ranged from 20 to 100 adults, most commonly aged 18–55, and were predominantly women with Fitzpatrick skin types II–V, most commonly type IV. Only three studies included male participants [15, 16, 17]. Study populations were drawn from Egypt, Iran, Brazil, India, China, and Pakistan [13, 14, 15, 16, 17, 18, 19, 20].

### 3.4 Melasma Area and Severity Index (MASI) Scores

Seven of eight studies used MASI or mMASI as the primary endpoint, consistently demonstrating significant pigment reduction with microneedling adjunct to TXA [13, 14, 15, 16, 17, 18, 19]. Ebrahim et al. and Poostiyan et al. found similar improvements between microneedling and microinjection groups [13, 19]. While Sharma et al. observed equal efficacy with oral versus intradermal TXA administration [17]. Cassiano et al. found that microneedling, oral TXA, and their combination all produced significant improvements in mMASI compared to control; however, combination therapy offered no additional benefit over either modality alone. At follow‐up, microneedling with or without TXA maintained improvement, whereas oral TXA alone demonstrated the poorest durability, with outcomes worsening by Day 120 [18].

Raza et al. compared microneedling with TXA versus vitamin C in a split‐face design and found earlier improvement with TXA after one session; however, after three treatments (6 weeks), both treatments produced similar mMASI reductions. Neither modality was effective for deep dermal melasma [15]. Debasmita et al. found that microneedling and Q‐switched Nd:YAG laser, when combined with topical 3% TXA gel, produced comparable reductions in mMASI, with no significant difference between the two modalities [16]. Arida et al. reported no significant difference in hemi‐MASI improvement between microneedling with TXA versus saline, with both sides showing similar reductions over 16 weeks [14].

### 3.5 Objective Assessments

Xu et al. found that microneedling with TXA produced greater clinical improvement than topical TXA alone in a split‐face design, with superior blinded physician ratings by Week 12 [20]. VISIA imaging similarly showed greater reduction in brown‐spot severity with microneedling‐based TXA, and Mexameter analysis demonstrated a significantly larger decrease in melanin index compared with topical TXA alone. Other skin biophysical parameters such as skin hydration, transepidermal water loss, roughness, erythema index, and elasticity did not differ between the two groups [20].

### 3.6 Patient and Clinician Satisfaction

Ebrahim et al. and Poostiyan et al. both reported higher patient satisfaction with microneedling‐based TXA delivery compared with intradermal microinjections [13, 19]. Xu et al. also found higher satisfaction with microneedling plus TXA than with topical TXA alone [20].

In contrast, Arida et al. observed no difference in patient or physician satisfaction between microneedling with TXA versus saline [14]. Raza et al. similarly found comparable patient and physician satisfaction between microneedling with vitamin C and microneedling with TXA after one session [15]. Debasmita et al. reported no significant difference in patient satisfaction between TXA combined with microneedling versus Q‐switched Nd:YAG laser [16].

### 3.7 Adverse Effects

Adverse effects across studies were generally mild and transient. Microneedling and intradermal injection commonly caused short‐lived erythema and edema, with irritation more frequent after microneedling and injection‐site pain more common with microinjections [13, 19]. Debasmita et al. also reported erythema, pain, and burning with TXA combined with Q‐switched Nd:YAG laser or microneedling, with pain and erythema more frequent in the microneedling group [16]. Rare events with microneedling included post‐inflammatory hyperpigmentation [13] and herpes simplex reactivation [18]. Oral TXA was associated with systemic effects such as gastrointestinal upset, headache, and hypomenorrhea, leading to one treatment discontinuation [18].

### 3.8 Durability of Treatment Responses

Sharma et al. reported relapse only in the oral TXA group at 24 weeks, with no recurrence in those receiving intradermal TXA microinjections [17]. Ebrahim et al. found similar recurrence rates of 16% for both microneedling and microinjection groups [19]. Cassiano et al. showed that microneedling, microneedling with TXA, and oral TXA all maintained similar improvements at 60 days; however, by day 120, oral TXA alone demonstrated the poorest durability with outcomes worse than control. Both microneedling and microneedling with TXA maintained similar improvements at day 120 [18].

## 4 Discussion

The findings of this scoping review indicate that microneedling‐based TXA delivery demonstrated clinical effectiveness comparable to other established treatment approaches, while offering potential advantages in patient satisfaction, tolerability, and durability of response. When evaluated collectively, these studies suggest that localized delivery through microneedling may mitigate some limitations associated with systemic or injectable therapy, particularly with respect to adverse effects and relapse. This perspective emerges from comparison across heterogeneous studies and may help inform individualized treatment selection in clinical practice.

### 4.1 Objective Outcomes

Across the included trials, objective outcome measurements consistently supported the effectiveness of TXA delivery through microneedling for melasma. MASI and mMASI were the most frequently used endpoints, both of which are clinical scoring systems designed to quantify melasma severity. MASI incorporates assessments of lesion area, darkness, and homogeneity, while mMASI excludes homogeneity to reduce subjectivity [21]. However, both rely on clinical judgment, making them semi‐quantitative and susceptible to interobserver variability [21, 22]. Significant reductions in MASI and mMASI were reported in nearly all studies evaluating microneedling with TXA, although improvements were generally comparable to oral, topical, or intradermal TXA delivery methods or to microneedling alone [13, 14, 15, 16, 17, 18, 19].

More objective device‐based assessments were incorporated only by Xu et al., who used VISIA imaging and Mexameter analysis to quantify changes in pigmentation [20, 23, 24, 25]. VISIA, a high‐resolution facial imaging system, provided objective brown‐spot severity assessments [20, 24], while the Mexameter measured pigment changes through a melanin index [20, 25]. Both tools demonstrated greater pigment improvement with microneedling‐based TXA compared with topical TXA alone at 12 weeks, further supported by blinded clinical evaluations favoring the microneedling‐TXA side [20]. These findings highlight the potential added value of microneedling for TXA delivery when assessed with more objective tools.

### 4.2 Subjective Outcomes

Subjective outcomes further highlight the potential advantages of microneedling‐based TXA for treatment of melasma. In multiple trials, patients reported greater satisfaction with microneedling‐based TXA than with intradermal TXA injections or topical TXA alone [13, 19, 20]. These differences likely reflect both the greater clinical improvements observed with microneedling‐based TXA along with the procedural advantages of microneedling itself, including its minimally invasive nature, reduced discomfort, and overall greater tolerability.

However, not all studies showed subjective advantages. Arida et al. and Raza et al. found no significant differences in patient or clinician satisfaction between microneedling with TXA and microneedling with saline or vitamin C [14, 15]. Similarly, Debasmita et al. reported comparable satisfaction between TXA combined with microneedling and TXA combined with Q‐switched Nd:YAG laser [16]. These findings suggest that the patient perceived benefits may be derived from the procedural effects of microneedling itself rather than from the addition of TXA.

### 4.3 Durability

Durability data was limited; however, suggested differences between delivery methods. Sharma et al. reported relapse only in the oral TXA group at 24 weeks, with no recurrence following intradermal microinjections [17]. Ebrahim et al. similarly reported comparable recurrence rates for microneedling and microinjection groups [19]. Cassiano et al. reported microneedling, with or without TXA, maintained improvement through Day 120, whereas oral TXA demonstrated the poorest durability, with outcomes worsening relative to control [18]. Collectively, these findings suggest that localized TXA delivery through microneedling may offer more sustained results than systemic therapy.

### 4.4 Safety and Tolerability

Microneedling‐based TXA delivery demonstrated a favorable safety profile across all studies, with most adverse effects being mild and transient, such as erythema, edema, and irritation [13, 16, 19]. Although Debasmita et al. noted greater erythema and pain with microneedling compared to Q‐switched Nd:YAG laser, these reactions resolved without interventions [16]. Rare events, including post‐inflammatory hyperpigmentation [13] and herpes simplex reactivation [18], highlight the importance of identifying susceptible patients, such as individuals with darker skin or a history of herpes simplex infection, before initiating microneedling.

Compared with intradermal injections, microneedling was consistently better tolerated, which may contribute to its higher patient satisfaction despite similar efficacy. In contrast, oral TXA was associated with systemic effects, such as gastrointestinal discomfort, headache, and hypomenorrhea, occasionally leading to discontinuation [18]. Collectively, these findings support microneedling as a well‐tolerated, minimally invasive option for patients who may not tolerate systemic or injectable TXA.

### 4.5 Limitations of Evidence

This review has several limitations. MASI and mMASI, the primary endpoints across studies, are only semi‐objective and prone to interobserver variability [21, 22]. Considerable heterogeneity existed in microneedling protocols, TXA concentrations, treatment intervals, comparators, and follow‐up durations, limiting cross‐study comparability [13, 14, 15, 16, 17, 18, 19, 20]. Only three studies included longer‐term follow‐up [17, 18, 19]. Additionally, one study evaluated oral versus intradermal TXA rather than microneedling, complicating comparisons [17]. Collectively, these limitations highlight the need for larger, standardized, and longer‐term trials that incorporate more objective assessment modalities to better clarify the role of microneedling with TXA in the treatment of melasma.

## 5 Conclusion

In conclusion, evidence from eight clinical trials demonstrates that TXA delivered through microneedling is a safe, effective, and well‐tolerated treatment option for melasma [13, 14, 15, 16, 17, 18, 19, 20]. While outcomes are comparable to other routes of TXA administration and to comparator therapies, this scoping review highlights patient‐centered advantages in tolerability and durability that may inform individualized treatment selection. Future studies should incorporate standardized treatment protocols, objective outcome measures, and extended follow‐up to better define long‐term effectiveness.

## Author Contributions

All authors contributed to the conception, drafting, and critical revision of the manuscript and approved the final version.

## Funding

The authors have nothing to report.

## Ethics Statement

The authors have nothing to report.

## Conflicts of Interest

The authors declare no conflicts of interest.

## Data Availability

The authors have nothing to report.

## References

[1] A. C. Handel , L. D. Miot , and H. A. Miot , “Melasma: A Clinical and Epidemiological Review,” Anais Brasileiros de Dermatologia 89, no. 5 (2014): 771–782, 10.1590/abd1806-4841.20143063. 25184917 PMC4155956

[2] W. Chen , Y. Wan , Y. Sun , C. Gao , and J. Li , “Prevalence of Depression in Melasma: A Systematic Review and Meta‐Analysis,” Frontiers in Psychiatry 14 (2024): 1276906, 10.3389/fpsyt.2023.1276906. 38260775 PMC10800906

[3] Y. Zhu , X. Zeng , J. Ying , Y. Cai , Y. Qiu , and W. Xiang , “Evaluating the Quality of Life Among Melasma Patients Using the MELASQoL Scale: A Systematic Review and Meta‐Analysis,” PLoS One 17, no. 1 (2022): e0262833, 10.1371/journal.pone.0262833. 35085327 PMC8794204

[4] G. Dabas , K. Vinay , D. Parsad , A. Kumar , and M. S. Kumaran , “Psychological Disturbances in Patients With Pigmentary Disorders: A Cross‐Sectional Study,” Journal of the European Academy of Dermatology and Venereology: JEADV 34, no. 2 (2020): 392–399, 10.1111/jdv.15987. 31566833

[5] V. K. Mahajan , A. Patil , L. Blicharz , et al., “Medical Therapies for Melasma,” Journal of Cosmetic Dermatology 21, no. 9 (2022): 3707–3728, 10.1111/jocd.15242. 35854432

[6] R. Sarkar , E. B. Handog , A. Das , et al., “Topical and Systemic Therapies in Melasma: A Systematic Review,” Indian Dermatology Online Journal 14, no. 6 (2023): 769–781, 10.4103/idoj.idoj_490_22. 38099013 PMC10718129

[7] P. E. Grimes , J. Bhawan , I. L. Guevara , et al., “Continuous Therapy Followed by a Maintenance Therapy Regimen With a Triple Combination Cream for Melasma,” Journal of the American Academy of Dermatology 62, no. 6 (2010): 962–967, 10.1016/j.jaad.2009.06.067. 20398959

[8] K. Maeda and Y. Tomita , “Mechanism of the Inhibitory Effect of Tranexamic Acid on Melanogenesis in Cultured Human Melanocytes in the Presence of Keratinocyte‐Conditioned Medium,” Journal of Health Science 53, no. 4 (2007): 389–396, 10.1248/jhs.53.389.

[9] L. Zhang , W. Q. Tan , Q. Q. Fang , et al., “Tranexamic Acid for Adults With Melasma: A Systematic Review and Meta‐Analysis,” BioMed Research International 2018 (2018): 1683414, 10.1155/2018/1683414. 30533427 PMC6247725

[10] A. J. M. Bailey , H. O. Li , M. G. Tan , W. Cheng , and J. S. Dover , “Microneedling as an Adjuvant to Topical Therapies for Melasma: A Systematic Review and Meta‐Analysis,” Journal of the American Academy of Dermatology 86, no. 4 (2022): 797–810, 10.1016/j.jaad.2021.03.116. 33857549

[11] A. Singh and S. Yadav , “Microneedling: Advances and Widening Horizons,” Indian Dermatology Online Journal 7, no. 4 (2016): 244–254, 10.4103/2229-5178.185468. 27559496 PMC4976400

[12] R. Hamed , B. J. Abu Nahia , A. Z. Alkilani , Y. Al‐Adhami , and R. Obaidat , “Recent Advances in Microneedling‐Assisted Cosmetic Applications,” Cosmetics 11, no. 2 (2024): 51, 10.3390/cosmetics11020051.

[13] N. Poostiyan , M. Alizadeh , Z. Shahmoradi , and F. Fatemi Naeini , “Tranexamic Acid Microinjections Versus Tranexamic Acid Mesoneedling in the Treatment of Facial Melasma: A Randomized Assessor‐Blind Split‐Face Controlled Trial,” Journal of Cosmetic Dermatology 22, no. 4 (2023): 1238–1244, 10.1111/jocd.15580. 36606390

[14] D. Kuster Kaminski Arida , P. R. Orso Rebellato , G. L. de Marioto Campos , et al., “Randomized, Double‐Blind, Placebo‐Controlled Split‐Face Trial of the Efficacy of Tranexamic Acid by Drug Delivery Through Microneedling in the Treatment of Melasma,” Journal of Cosmetic Dermatology 20 (2021): 4005–4010, 10.1111/jocd.14257. 34077619

[15] M. H. Raza , N. Iftikhar , A. Anwar , A. A. Mashhood , S. Tariq , and M. A. B. Hamid , “Split‐Face Comparative Analysis of Micro‐Needling With Tranexamic Acid vs Vitamin C Serum in Melasma,” Journal of Ayub Medical College, Abbottabad 34, no. 1 (2022): 169–172, 10.55519/jamc-01-9840. 35466647

[16] B. Debasmita , C. Raj , A. Ishan , and D. Ipsita , “A Prospective Randomized Controlled Trial of Q‐Switched Nd:YAG Laser With Topical 3% Tranexamic Acid (TA) Versus Microneedling With Topical 3% Tranexamic Acid (TA) in Treatment of Melasma,” Journal of Cosmetic Dermatology 21 (2021): 2801–2807, 10.1111/jocd.14532. 34636493

[17] R. Sharma , V. K. Mahajan , K. S. Mehta , P. S. Chauhan , R. Rawat , and T. N. Shiny , “Therapeutic Efficacy and Safety of Oral Tranexamic Acid and That of Tranexamic Acid Local Infiltration With Microinjections in Patients With Melasma: A Comparative Study,” Clinical and Experimental Dermatology 42, no. 7 (2017): 728–734, 10.1111/ced.13164. 28649780

[18] D. Cassiano , A. C. C. Esposito , K. Hassun , et al., “Efficacy and Safety of Microneedling and Oral Tranexamic Acid in the Treatment of Facial Melasma in Women: An Open, Evaluator‐Blinded, Randomized Clinical Trial,” Journal of the American Academy of Dermatology 83, no. 4 (2020): 1176–1178, 10.1016/j.jaad.2020.02.002. 32035945

[19] H. M. Ebrahim , A. Said Abdelshafy , F. Khattab , and K. Gharib , “Tranexamic Acid for Melasma Treatment: A Split‐Face Study,” Dermatologic Surgery 46, no. 11 (2020): e102–e107, 10.1097/DSS.0000000000002449. 32701529

[20] Y. Xu , R. Ma , J. Juliandri , et al., “Efficacy of Functional Microarray of Microneedles Combined With Topical Tranexamic Acid for Melasma,” Medicine 96, no. 19 (2017): e6897, 10.1097/md.0000000000006897. 28489798 PMC5428632

[21] A. G. Pandya , L. S. Hynan , R. Bhore , et al., “Reliability Assessment and Validation of the Melasma Area and Severity Index (MASI) and a New Modified MASI Scoring Method,” Journal of the American Academy of Dermatology 64, no. 1 (2011): 78–83.e2, 10.1016/j.jaad.2009.10.051. 20398960

[22] K. Heidemeyer , S. Cazzaniga , L. Feldmeyer , et al., “Skin Hyperpigmentation Index in Melasma: A Complementary Method to Classic Scoring Systems,” Journal of Cosmetic Dermatology 22, no. 12 (2023): 3405–3412, 10.1111/jocd.15866. 37349912

[23] Y. W. Huang , W. Arkesteijn , Y. J. Lai , and C. Y. Ng , “A Comparative Study of an Advanced Skin Imaging System in Diagnosing Facial Pigmentary and Inflammatory Conditions,” Scientific Reports 14, no. 1 (2024): 14673, 10.1038/s41598-024-63274-7. 38918427 PMC11199608

[24] P. Zawodny , E. Stój , P. Kulig , K. Skonieczna‐Żydecka , and J. Sieńko , “Visia Skin Analysis System as a Tool to Evaluate the Reduction of Pigmented Skin and Vascular Lesions Using the 532 Nm Laser,” Clinical, Cosmetic and Investigational Dermatology 15 (2022): 2187–2195, 10.2147/ccid.s380388. 36267688 PMC9578358

[25] S. Vasudevan , W. C. Vogt , S. Weininger , and T. Joshua Pfefer , “Melanometry for Objective Evaluation of Skin Pigmentation in Pulse Oximetry Studies,” Communications Medicine 4, no. 1 (2024): 138, 10.1038/s43856-024-00550-7. 38992188 PMC11239860

